# A Systematic Review and Meta-Analysis of Single-Dose GnRH Agonist on the Day of Frozen Embryo Transfer in Artificial Cycles: Preliminary Evidence from Randomized Trials

**DOI:** 10.3390/jcm14165763

**Published:** 2025-08-14

**Authors:** Luz Franco Pire, Laura Morales López, María Hernández Hernández, Raquel Campos Romero, Ignacio Cristóbal García, Ignacio Cristóbal Quevedo

**Affiliations:** 1 Department of Gynecology, Hospital Clínico San Carlos, 28040 Madrid, Spain; 2 Department of Gynecology, Hospital San Pedro de Alcántara, 10003 Cáceres, Spain; 3 Department of Gynecology, Faculty of Medicine, Universidad Complutense de Madrid, 28040 Madrid, Spain

**Keywords:** frozen embryo transfer, GnRH agonists, luteal support, artificial cycles

## Abstract

**Background/Objectives**: GnRH agonists may offer potential benefits when used for luteal phase support in assisted reproductive treatments. This systematic review and meta-analysis of randomized controlled trials evaluates the effect of a single-dose administration of gonadotropin-releasing hormone (GnRH) agonist on the day of frozen-thawed embryo transfer (FET) in artificial cycles, in terms of reproductive outcomes. **Methods**: A comprehensive literature search was performed using the PubMed and Cochrane databases to identify relevant studies. The outcomes assessed were live birth rate, clinical pregnancy rate, positive pregnancy test, implantation rate, and miscarriage rate. Three randomized controlled trials were included in the analysis. **Results**: The clinical pregnancy rate (56.5% vs. 47.4%; OR 1.27; 95% CI: 1.01–1.60; *p* = 0.0426) and live birth rate (34.3% vs. 23.9%; OR 1.71; 95% CI: 1.00–2.91; *p* = 0.0483) were significantly higher in the treatment group compared to the control group. No statistically significant differences were observed between the groups in terms of positive pregnancy test, implantation rate, or miscarriage rate, although the analysis revealed a trend toward improved outcomes in the intervention group. **Conclusions**: In summary, although our meta-analysis indicates that a single dose of GnRH agonist in artificial FET cycles may be associated with improved clinical pregnancy and live birth rates, these findings are based on a limited number of available trials. Larger, well-designed randomized controlled trials are urgently needed before any changes to clinical recommendations can be justified.

## 1. Introduction

The number of frozen embryo transfer (FET) cycles performed in assisted reproductive technology (ART) has noticeably increased in the past years, now accounting for more than 60% of autologous transfers in many countries, with a parallel increase in the adoption of the ‘freeze-all’ strategy and single embryo transfer, which has contributed to the reduction of multiple pregnancies and their associated complications [1]. This has been possible largely due to advancements in embryo freezing protocols and improvements in cryopreservation techniques, as it has demonstrated embryo survival rates of over 95%, with better clinical and neonatal outcomes compared to slow freezing, and has enabled the expansion of fertility preservation and the use of preimplantation genetic testing [2,3]. Vitrification enables the preservation of surplus embryos within the current single embryo transfer policy and prevents the development of late-onset ovarian hyperstimulation syndrome in high-risk cases [4,5].

Compared to fresh embryo transfer (ET) cycles, current evidence [6] suggests that birth results do not differ significantly among women who undergo FET, while vitrification makes possible the preservation of surplus embryos, thus increasing cumulative live-birth rates. In addition, it also enables physicians to practice a single embryo transfer (SET) as well as to perform preimplantation genetic testing. Moreover, as a consequence of this surge of FET cycles, there has been a decrease in late moderate to severe ovarian hyperstimulation syndrome compared with fresh embryo transfer cycles, making ART safer for patients. As a result, it has become increasingly common in ART to adopt the ‘freeze-all’ strategy, in which all embryos are cryopreserved for transfer in subsequent, unstimulated cycles. Although the ‘freeze-all’ strategy significantly reduces the risk of ovarian hyperstimulation syndrome, especially in high-response patients, the evidence synthesized by the Cochrane Gynaecology and Fertility Group indicates that there are no relevant differences in the cumulative live birth rate between FET and fresh transfer in the general population, and that the risk of hypertensive disorders of pregnancy and large for gestational age newborns may be higher after FET [6,7]. Therefore, the choice of strategy should be individualized according to the patient’s profile.

Despite the increased application of FET and the significant improvements in implantation and live birth rate resulting from this technique, the choice of the most optimal endometrium preparation protocol remains to be determined. It is well known that during the natural window of implantation, the endometrium undergoes through complex molecular and morphological changes that ensure that it is receptive for the moment the blastocyst interacts with the endometrium. However, administration of exogenous hormones may alter this interaction and lead to decrease implantation potential. Particularly in hormone replacement therapy (HRT) in delayed embryo transfers, the absence of a corpus luteum results in insufficient endogenous progesterone and other factors critical for implantation [8,9]. This is the reason endometrial preparation and adequate synchronization with embryo development represents a critical step in the final success of this technique. Therefore, choosing the most suitable protocol for endometrium preparation, as well as adequate hormonal support during the luteal phase, still remains a challenge.

Different FET clinical protocols have been proposed to prepare the endometrium, yet the best approach is not known. According to the strategy used for endometrial development, cycles can be classified as the following:

- Natural cycles (spontaneous ovulation) or modified natural cycles (spontaneous ovulation triggered with hCG or controlled with GnRH antagonists): Natural cycles have gained popularity, as they are considered a more physiological approach. Here, we rely on a woman’s spontaneous ovulation, and no medication is required. The main advantage of this method is that the endometrium develops under a natural hormonal environment with endogenous corpus luteum activity, which may offer benefits that are otherwise lost in artificial cycles (e.g., preservation of vasoactive progesterone produced by the corpus luteum, associated with lower risk of preeclampsia). However, its main limitation is logistical—it requires intensive monitoring, and there is a risk of cycle cancellation due to missed ovulation or poor timing for embryo transfer. This lack of control has led to the use of modified natural cycles, which include controlled ovulation and result in a better match between endometrium and embryo synchronization. In this case, spontaneous ovulation is triggered with hCG when the dominant follicle is between 16 and 20 mm in diameter, closely resembling natural cycles. Alternatively, ovulation is regulated with GnRH antagonists to suppress premature ovulation, which provides better control and increases the chances of successful implantation.
- Artificial or programmed cycles, also referred to as hormone replacement therapy (HRT) cycles): In this type of cycle, exogenous estrogen and progesterone are administered with or without prior pituitary suppression using GnRH agonists. These protocols enable adequate endometrial development while suppressing hypothalamic FSH secretion, thus preventing follicular recruitment.
- Mildly stimulated cycles, using low-dose gonadotropins or letrozole to promote follicular development and controlled ovulation.

In clinical practice, the most commonly used approaches are the natural and artificial (HRT) cycles. When it comes to decide whether to choose one or the other one, several aspects must be taken into consideration. As previously mentioned, natural cycles are based on a woman’s own ovulation, whereas artificial cycles simulate a luteal environment without ovulation. Artificial cycles are anovulatory and lack corpus luteum formation. Since the corpus luteum produces progesterone until approximately the 9th week of gestation—after which placental production predominates—proper luteal phase support is required until at least the 10th week of pregnancy (luteo-placental shift). This is essential not only to support implantation but also to maintain early pregnancy and reduce miscarriage rates [8,10].

Each has advantages and disadvantages. On the one hand, natural cycles relieve the patient from the burden of unnecessary medication while preserving endogenous corpus luteum function, but on the other hand, close monitoring is required, as well as an increased risk of cancellation due to timing issues. While these require more intensive monitoring and have a higher cancellation rates, recent randomized trials suggest that, in women with regular cycles, natural-cycle FET may result in higher live birth rates and lower miscarriage rates than HRT cycles [11]. In contrast, artificial cycles offer the convenience of scheduling and reduce the risk of cancellation, but they lack corpus luteum function and have been associated with altered obstetric outcomes, such as increased risk of hypertensive disorders of pregnancy. Women with regular menstrual cycles may be offered to carry out the embryo transfer after spontaneous ovulation, while women with irregular cycles or polycystic ovaries may not be suitable for this protocol, but clinicians should be aware of the increased maternal risks and consider individualized protocols and close monitoring [12].

Recently, the use of gonadotropin-releasing hormone agonists (GnRH-a) as luteal phase support has gained attention as a potential adjunct to improve endometrial receptivity and embryonic development. Several studies have proposed that administering GnRH-a during the implantation window may enhance reproductive outcomes, possibly through the stimulation of GnRH receptors expressed in both the endometrium and the embryo [13,14], or by inducing LH surges that support corpus luteum function in ovulatory cycles [15,16,17], though no standard dosing or indication has been established for this purpose in FET cycles [12].

There is growing evidence that GnRH agonist supplementation during the luteal phase can significantly improve implantation and clinical pregnancy rates in fresh embryo transfer cycles [18,19]. The literature indicates that adding a single or double dose of 0.1 mg subcutaneous GnRH agonist (typically on day 3 or days 3 and 6 post-embryo transfer) to standard vaginal progesterone support can increase clinical pregnancy rates in FET cycles, but the impact on live birth is less certain [20].

It has also been investigated whether GnRH agonists may exert direct effects on the endometrium. A study suggested that prior suppression with GnRH-a might upregulate implantation-related molecules (such as integrin αvβ3) or increase the number of endometrial pinopodes, potentially enhancing receptivity [16]. Additionally, in patients with underlying conditions, such as endometriosis or adenomyosis, suppression with GnRH agonists may reduce uterine estrogenic and inflammatory activity, thereby improving the implantation environment. In patients with recurrent implantation failure, retrospective data suggest a greater benefit from luteal GnRH agonist administration [16].

However, to date, few studies have specifically addressed the use of GnRH-a in FET cycles. Existing studies are heterogeneous in terms of endometrial preparation protocols (natural or modified natural vs. artificial), the number of GnRH-a doses administered, and the timing of administration. While some studies have shown significant benefits, particularly in natural cycles or in patients with prior implantation failure [21,22], others have not demonstrated statistically significant improvements [10,23]. This underscores the need for further research to determine the true clinical value of this strategy.

Although the present meta-analysis is restricted to randomized controlled trials (RCTs), several non-randomized studies and retrospective analyses have explored the administration of GnRH agonists during artificial FET cycles, with results that support a possible beneficial effect but also illustrate the ongoing controversy.

For example, Chang et al. [16] conducted a retrospective cohort study in patients undergoing GnRH agonist-supplemented HRT cycles and found an increased live birth rate with a single additional dose of GnRH agonist compared to standard luteal support. Jigal et al. [21] observed improved cycle outcomes in natural cycle FET with modified luteal support, including the use of GnRH agonists. However, other observational studies have produced conflicting results, and the lack of randomization, unmeasured confounders, and protocol heterogeneity must be recognized as significant limitations in these analyses [10,23]. Proposed mechanisms underlying the observed benefits of GnRH agonist supplementation in the luteal phase include several biological pathways:

- Endometrial receptivity modulation: GnRH receptors are present in the human endometrium. Agonist stimulation may upregulate key implantation molecules (e.g., integrin αvβ3, HOXA10), as shown in endometrial biopsies following GnRH-a use.
- Direct embryonic effects: GnRH receptors are also expressed on pre-implantation embryos; exposure to agonist in culture or in vivo may enhance embryonic development or hatching potential.
- Luteal function and local immune modulation: In ovulatory cycles, an additional GnRH-a bolus can trigger endogenous LH surges, supporting the corpus luteum and increasing progesterone and other luteotropic factors. While this effect is less relevant in artificial cycles (lacking corpus luteum), local effects on endometrial receptivity and immune environment may predominate.

Furthermore, certain high-risk populations (such as women with recurrent implantation failure, endometriosis or adenomyosis, as mentioned above) may derive particular benefit from luteal GnRH-a supplementation, as suggested by subgroup analyses and case series, but high-quality RCTs in these subgroups are lacking. Taken together, these retrospective and observational provide a biological rationale for the intervention and support ongoing research, but, as emphasized in our manuscript, robust randomized evidence remains insufficient for definitive recommendations.

Importantly, dedicated studies are needed to assess the effect of GnRH-a administration based on the type of endometrial preparation and the number of doses given. The impact of GnRH-a in artificial cycles, where there is no corpus luteum, may differ significantly from that in natural cycles. Additionally, dosing strategies and the cost-effectiveness of the intervention are important considerations that may influence outcomes and clinical decision-making. Key gaps include the lack of large, high-quality randomized trials powered for live birth, insufficient data on perinatal safety, and uncertainty regarding the best protocol for specific subgroups (e.g., endometriosis, adenomyosis, recurrent implantation failure).

In this context, the present study aims to systematically review the available evidence on the administration of a single dose of GnRH agonist on the day of frozen embryo transfer in artificial endometrial preparation cycles, evaluating its potential impact on reproductive outcomes and discussing its implications for current clinical practice. Due to the paucity of high-quality data, critical uncertainties remain regarding both efficacy and optimal protocols. Thus, any findings in the current literature, including this synthesis, must be regarded as hypothesis-generating.

## 2. Materials and Methods

A systematic review and meta-analysis has been conducted of all published studies that have evaluated the effect of administering a single dose of GnRH-a on the day of embryo transfer in artificial endometrial preparation cycles including as outcomes the interest implantation rate, positive pregnancy rate, live birth rate, clinical pregnancy rate, and miscarriage rate. This study was conducted in accordance with the PRISMA guidelines. This review has not been registered.

### 2.1. Eligibility Criteria

The present study incorporated all RCTs that directly compared the outcomes of interest between women receiving a single dose of GnRH-a on the day of embryo transfer and those managed with standard protocols or placebo in artificial endometrial preparation cycles. The primary outcomes evaluated included live birth rate (LBR), implantation rate, positive pregnancy test rate, and clinical pregnancy rate. The inclusion of studies in this analysis was contingent upon the reporting of at least one of the specified outcomes.

### 2.2. Exclusion Criteria

Randomized controlled trials were excluded if they involved the use of fresh embryos, as the objective of this study was to specifically evaluate frozen–thawed embryo transfer (FET) cycles. It is important to note that studies were excluded from consideration if they assessed multiple doses of GnRH-a, if the administration of the agonist occurred on a day other than the embryo transfer day, or if natural endometrial preparation cycles were used as an alternative to artificial protocols. Furthermore, non-randomized studies, retrospective analyses, case series, and conference abstracts were excluded in order to maintain methodological rigor and data reliability.

### 2.3. Search Strategy and Study Selection

A comprehensive online literature search was conducted using PubMed and the Cochrane Library (Cochrane Controlled Trials Register) from 2005 through April 2025. The following search terms were used: (frozen) OR (embryo transfer) OR (cryopreservation) AND (GnRH agonist) AND (luteal phase support). Additionally, a manual search was performed to identify further potentially eligible studies from the reference lists of relevant articles and systematic reviews. Previous meta-analyses were also reviewed and compared with our search to ensure the inclusion of all related studies.

Once the search was complete, Rayyan was employed to perform a systematic review. Prior to commencing the initial screening by title and abstract, duplicate articles were eliminated. Thereafter, records that did not meet the inclusion criteria were removed. The full texts of potentially eligible records were obtained through PubMed, the clinical trials (clinicaltrials.gov), and clinical trials registry (https://www.clinicaltrialsregister.eu/, accessed on 15 May 2025) databases. The eligibility of the trials was assessed independently and the reasons for exclusion documented. Disagreements between reviewers were resolved through discussion.

The risk of bias assessment was conducted using Revman 5.4, in accordance with the criteria defined in the Cochrane Handbook. The domains of random sequence generation, allocation concealment, blinding of participants and healthcare personnel, blinding of assessors, incomplete data, and selective reporting were classified as high risk, low risk, or uncertain risk. In the event that the data did not permit the determination of whether the risk is high or low, the study was classified as uncertain risk.

A study was considered to be of low risk of bias when all domains were classified as low risk, of uncertain risk when there was no high-risk domain and at least one domain was classified as uncertain risk, and of high risk when one domain was classified as high risk.

### 2.4. Statistical Analysis

All relevant data were extracted from the included studies and analyzed using Python statistical software (version number: Python 3.13.6). A fixed-effects model was employed for the meta-analysis, as the included studies exhibited a high degree of homogeneity with regard to cycle type (all artificial), GnRH-a dose, and timing of administration. The effect of the intervention in each eligible study was reported as an odds ratio (OR), and a 95% confidence interval (CI) was used to assess the precision of the estimates. Due to the small number of included studies (n = 3), formal assessment of publication bias (such as funnel plots or Egger’s test) was not performed, as these methods are unreliable with so few studies. Similarly, subgroup and sensitivity analyses were not feasible. The decision to employ a fixed-effects model was based on the low statistical heterogeneity observed (I^2^ = 0%) and on the clinical and methodological similarity of the included trials. We acknowledge that this approach inherently limits the exploration of clinical heterogeneity and underlying sources of bias. This methodological approach permitted a synthesis of the current available evidence and facilitated interpretation of the pooled results.

## 3. Results

**Figure 1:**
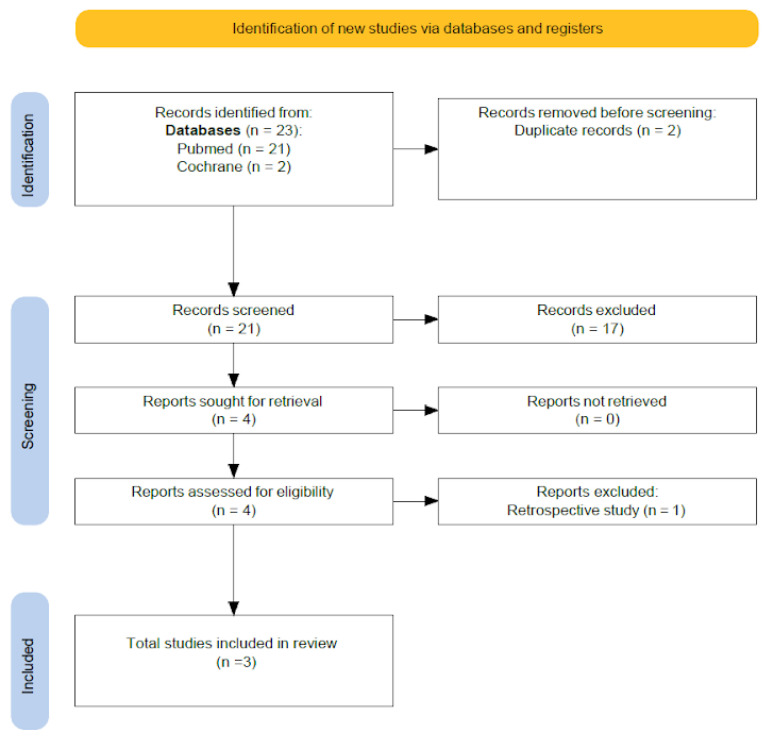
PRISMA flow diagram.

Initially, 21 potentially eligible articles were identified through the search strategy. After screening titles and abstracts, and subsequently reviewing the full texts, only four studies met all the predefined inclusion criteria. Of these four, one was excluded for being a retrospective cohort study rather than a randomized controlled trial. Ultimately, three randomized controlled trials were included in the final analysis. The process of study selection and reasons for exclusion at each stage are summarized in a PRISMA flow diagram (Figure 1), outlining the number of records identified, screened, excluded, and included in the final analysis. Although all studies employed artificial endometrial preparation, the luteal phase support protocols varied in terms of progesterone administration (route, dosage, and duration). Notably, one study, Wang et al. [23], did not fully specify the details of the luteal support provided. This clinical heterogeneity is a potential source of bias and may influence the overall results. Not all studied outcomes were available in every trial; for example, analyses of live birth rate and positive pregnancy test rate were limited to two out of three studies, further reducing power and the robustness of pooled estimates.

The main characteristics of the studies included in the meta-analysis are summarized in Table 1.

### 3.1. Implantation Rate

**Figure 2:**
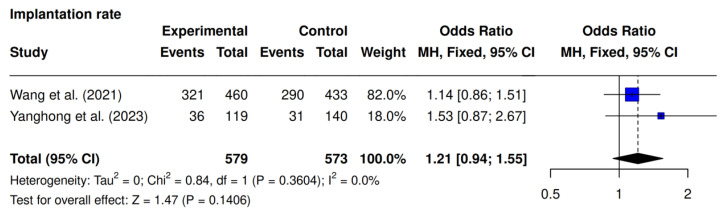
Comparative data analysis of implantation rate across the two studies included in the meta-analysis [9,23].

Two of the three studies, Wang et al. [23] and Yanghong et al. [9], assessed implantation rate. The pooled analysis demonstrated a tendency towards increased implantation in the intervention group in comparison with the control group (61.6% [357/579] vs. 56% [321/573]; OR 1.21; 95% CI 0.94–1.55; *p* = 0.14; I^2^ = 0%), although this did not reach statistical significance (Figure 2).

### 3.2. Positive Pregnancy Test Rate

**Figure 3:**
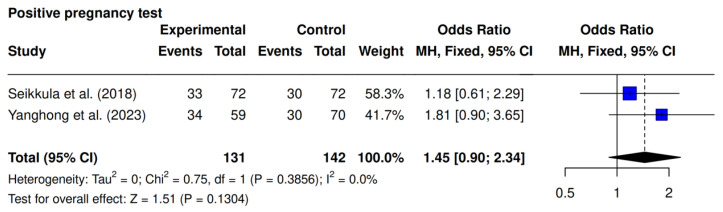
Comparative data analysis of positive pregnancy rate across the two studies included in the meta-analysis [9,10].

The present outcome was the focus of two studies, Seikkula et al. [10] and Yanghong et al. [9]. A higher rate was observed in the intervention group (51% [67/131] vs. 42% [60/142]; OR 1.45; 95% CI 0.90–2.34; *p* = 0.13; I^2^ = 0%), though it was not statistically significant (Figure 3).

### 3.3. Live Birth Rate (LBR)

**Figure 4:**
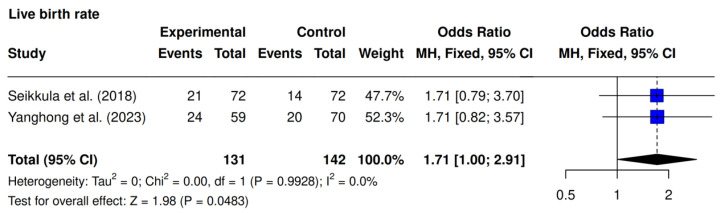
Comparative data analysis of live birth rate across the two studies included in the meta-analysis [9,10].

Two studies, Seikkula et al. [10] and Yanghong et al. [9], assessed LBR. The meta-analysis revealed a significantly higher LBR in the intervention group (34.3% [45/131] vs. 23.9% [34/142]; OR 1.71; 95% CI 1.00–2.91; *p* = 0.0483; I^2^ = 0%). (Figure 4).

### 3.4. Clinical Pregnancy Rate

**Figure 5:**
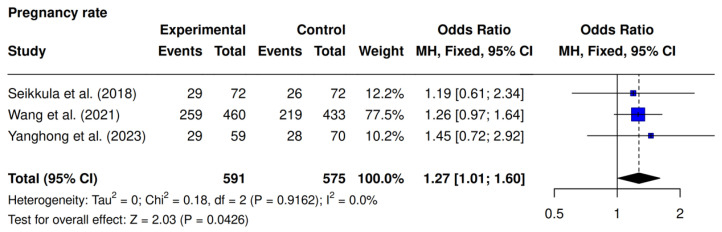
Comparative data analysis of clinical pregnancy rate across the three studies included in the meta-analysis [9,10,23].

Three studies were included [9,10,23]. The intervention group demonstrated significantly higher clinical pregnancy rates compared to the controls (56.5% [317/561] vs. 47.4% [272/575]; OR 1.27; 95% CI 1.01–1.60; *p* = 0.0426; I^2^ = 0%) (Figure 5).

### 3.5. Miscarriage Rate

**Figure 6:**
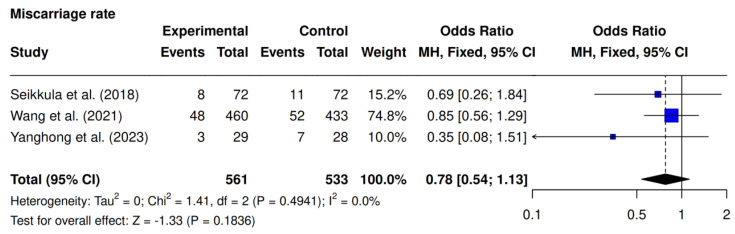
Comparative data analysis of miscarriage rate across the three studies included in the meta-analysis [9,10,23].

Three studies assessed miscarriage rates [9,10,23]. No significant difference was observed (10.5% [59/561] vs. 13.1% [70/533]; OR 0.78; 95% CI 0.54–1.13; *p* = 0.1836; I^2^ = 0%) (Figure 6).

## 4. Discussion

This meta-analysis included the three most recent studies evaluating the administration of a single dose of GnRH-a on the day of frozen embryo transfer as luteal phase support, encompassing 1094 patients.

The findings suggest a beneficial effect on clinical pregnancy and live birth rates, without conclusive improvements in implantation rate, positive pregnancy test rate, or miscarriage reduction.

Regarding clinical pregnancy rate, the analysis demonstrated a statistically significant improvement in the intervention group (OR 1.27; 95% CI 1.01–1.60; *p* = 0.0426), consistent with the meta-analysis by Pongpawan et al., which also reported an increase (52.05% vs. 47.29%; *p* = 0.04; RR = 1.09; 95% CI 1.00–1.18) [5]. Notably, Pongpawan found this effect more pronounced in natural cycles, whereas our results, focusing solely on artificial cycles, also indicate a benefit. The most recent meta-analysis established that the incorporation of 0.1 mg of subcutaneous GnRH agonist, administered as a single dose (typically on day 3 post-embryo transfer) or as a double dose (days 3 and 6), to standard vaginal progesterone support, was associated with enhanced clinical pregnancy rates (RR 1.86, 95% CrI 1.18–2.93) in FET cycles [20], as observed in our own research, though this meta-analyses added observational studies to its protocol.

The live birth rate was significantly higher in the intervention group (OR 1.71; 95% CI 1.00–2.91; *p* = 0.0483), consistent with the findings of Chang et al., who observed a significant increase in artificial cycles (OR 2.03; 95% CI 1.20–3.45; *p* = 0.009) [15]. Liu et al. also reported a 12.1% increase in the live birth rate (40.7% vs. 28.6%), though not statistically significant due to a limited sample size and statistical power [9]. Conversely, Wang et al. reported no significant differences, potentially due to variations in the route of luteal phase support administration [22]. Additionally, Wang et al. found no significant difference, which may be attributable to differences in the route or regimen of luteal phase support [9].

Meta-analyses in both fresh and frozen cycles suggest that GnRH-a supplementation may improve live birth and clinical pregnancy rates, but the quality of evidence is limited by heterogeneity in study design, patient selection, and protocol standardization [15,19]. The benefit appears more pronounced in certain subgroups, such as those with recurrent implantation failure or in artificial cycles, but this requires further validation [16].

Safety data is limited. Most studies did not systematically report perinatal or neonatal outcomes, and there is insufficient evidence to confirm the safety of this intervention for mother and child [9], as such, we could not study these variables.

No significant differences were observed in implantation rates or positive pregnancy test rates (OR 1.21; 95% CI 0.94–1.55, *p* = 0.14 and OR 1.45; 95% CI 0.90–2.34, *p* = 0.13, respectively). However, a slight improvement in both outcomes was noted in the intervention group, underscoring the need for larger studies. A number of studies and meta-analyses have reported improvements in clinical pregnancy and live birth rates with GnRH agonist supplementation. However, the lack of significant effect on implantation and biochemical pregnancy rates suggests that the mechanism of benefit may occur after implantation, potentially by supporting luteal function or early placental development rather than enhancing initial embryo–endometrial interaction [16,20].

Regarding miscarriage rates, a non-significant decrease was found in the intervention group (OR 0.78; 95% CI 0.54–1.13; *p* = 0.1836), in line with the findings of Seikkula et al., who reported a 14.7% reduction without statistical significance [10]. However, the power to detect differences in miscarriage rates is often limited by sample size, as miscarriage is a less frequent outcome compared to clinical pregnancy or live birth. Many studies are underpowered for this endpoint, and confidence intervals are wide, indicating imprecision [9]. Furthermore, subgroup analyses may reveal differential effects. For example, Ye et al. found improved implantation rates in women aged 35–37, but no significant difference in miscarriage rates overall, suggesting that any potential benefit of GnRH-a supplementation may be limited to specific populations or age groups [24].

Of particular note is the absence of statistical heterogeneity in the present study across all five outcomes (I^2^ = 0%), which lends support to the robustness and reliability of the findings. This finding indicates that the results are consistent across the included studies and that the observed effects are unlikely to be attributable to random variation or methodological differences. This homogeneity serves to reinforce the validity of the pooled estimates, thereby suggesting that the intervention effect remains stable within the context of analysis. Nevertheless, it is important to acknowledge several limitations. The dearth of included studies is particularly salient, as it curtails the generalizability of the conclusions and may circumscribe the statistical power to discern subtle variations or infrequent adverse outcomes. Several outcomes analyzed in this meta-analysis, including live birth rates and positive pregnancy test rates, were reported in only two of the three included studies, further limiting the statistical power and reliability of pooled estimates for these endpoints. This meta-analysis is limited by the small number of eligible randomized controlled trials and the total sample size, which restrict the power to detect modest treatment effects and reduces the precision of effect estimates. Additionally, the use of a fixed-effects model, while statistically justified by the lack of heterogeneity (I^2^ = 0%), does not account for potential clinical or methodological variability that may exist between studies. The inability to perform subgroup or sensitivity analyses, or to formally assess publication bias, further limits the strength of our conclusions. Notably, differences in luteal support regimens among the included studies add clinical heterogeneity that may influence outcomes. These limitations mean that our findings should be viewed as hypothesis-generating, rather than definitive guidance for clinical practice. Consequently, while the present findings are encouraging, further large-scale, well-designed randomized controlled trials are required to confirm these results and to clarify the optimal patient populations and protocols for the use of GnRH agonists in artificial FET cycles.

## 5. Conclusions

In summary, the findings of this study suggest that the administration of a single dose of GnRH agonist during the luteal phase in artificial frozen embryo transfer (FET) cycles may offer a significant clinical benefit, particularly by increasing clinical pregnancy and live birth rates. While improvements in positive pregnancy test rates, implantation rates, and miscarriage rates did not reach statistical significance, a consistent trend favoring the intervention group was observed across these secondary outcomes.

In conclusion, while our systematic review and meta-analysis summarize the current evidence regarding the use of single-dose GnRH agonist on the day of FET in artificial cycles, the strength and generalizability of the findings are restricted by the limited evidence base and methodological constraints. Additional large, high-quality, randomized trials are essential to confirm or refute these preliminary observations before any routine clinical application can be recommended.

## Figures and Tables

**Table 1:** Control trials included in our meta-analysis and their main characteristics. Details of luteal support regimens were not fully harmonized across studies; see text for implications regarding clinical heterogeneity.

Study Year	Patient Population	Protocolo FET	Luteal Pase Support + GnRH-a	Results
Seikkula et al. (2018) [10]	- Excluded women > 42, chromosomal abnormality, testicular or donated sperm without female cause of infertility, donated oocytes, congenital uterine anomalies, intramural myomas > 4 cm, submucous myoma, endometrial polyp > 1 cm, endometrium thickness < 6 mm before embryo transfer, untreated thyroid dysfunction hyperprolactinemia, allergy to triptorelin.	- Artificial cycle - Blastocyst embryos	- Triptorelin 0.1 mg at age 6 days of the transferred embryos - Micronized progesterone 600 mg vaginally	LBR: 29.2% vs. 19.4%, *p =* 0.11 CPR: 40.3% vs. 36,1%, *p =* 0.693 PPR: 45.8% vs 41.7%, *p* = 0.691 Misc. rate: 27.6% vs. 42.3%, *p =* 0.535
Wang et al. (2021) [23]	- Excluded women with infertility > 10 years, chromosomal abnormalities, hydrosalpinx, uterine malformations, submucosal myoma, history of tuberculosis or any uncontrolled endocrine disorder that may affect pregnancy, history of endometrial hyperplasia.	- Recruited only artificial cycles - Blastocyst embryos	- Triptorelin 0.1 mg on the day of transfer - Other luteal support not clarified	CPR: 56.3% vs. 50.58%, *p =* 0.086 Imp. rate: 39.98% vs. 38.01%, *p =* 0.425 Misc. Rate: 10.43% vs. 12.01%, *p =* 0.46
Yanghong Liu 2023 [9]	Exclusion criteria included females over 40 years of age or follicle-stimulating hormone (FSH) ≥ 20 IU/L, uterine anomalies, intramural myomas (≥4 cm), submucous fibroids, endometrium thickness less than 7 mm before embryo transfer, patients who had untreated systemic or endocrine disorders, such as diabetes mellitus, thyroid dysfunction, or hyperprolactinemia and female or male chromosomal abnormality.	- Artificial cycle - Blastocyst embryos	- Triptorelin 0.1 mg on the day of transfer - Progesterone injections (total dose of 80 mg)	CPR: 49.2% vs. 40%, *p* = 0.374 LBR: 40.7% vs. 28.3%, *p* = 0.208 PPT 57.6% vs. 42.9%, *p* = 0.095 Implantation rate 30.3% vs 22.1%, *p =* 0.138 Misc rate 10.3% vs. 25%, *p =* 0.269

## Data Availability

The data that support the findings of this study are not publicly available. Interested researchers may request access to the datasets by contacting the corresponding author via email.

## Abbreviations

The following abbreviations are used in this manuscript:

FET	Frozen embryo transfer
GnRHa	Gonadotropin-releasing hormone agonist
LBR	Live birth rate
HRT	Hormone replacement therapy
